# Disparities in sexual and reproductive health services utilization among urban and rural adolescents in southern Ethiopia, 2020: a comparative cross-sectional study

**DOI:** 10.1186/s12889-022-12634-x

**Published:** 2022-01-31

**Authors:** Aklilu Habte, Samuel Dessu, Biruk Bogale, Lire Lemma

**Affiliations:** 1 School of Public Health, College of Medicine and Health Sciences, Wachemo University, Hosanna, Ethiopia; 2 Department of Public Health, College of Medicine and Health Sciences, Wolkite University, Wolkite, Ethiopia; 3 Department of Epidemiology and Biostatistics, School of Public Health, College of Medicine and Health Sciences, Mizan Tepi University, Mizan Aman, Southern Ethiopia Ethiopia

## Abstract

**Background:**

Although studies on the uptake of Adolescent sexual and reproductive health (ASRH) services in Ethiopia have been conducted they have failed to show the disparity in service uptake among rural and urban settings. Once the extent and determinants of ASRH service uptake in urban and rural contexts are known, it will be crucial to provide evidence-based information and recommendations for potential interventions to reduce the burden of disease and disability among adolescents. This study aimed at determining the level of SRH service utilization among urban and rural adolescents in the Guraghe zone, Southern Ethiopia.

**Methods:**

A community-based comparative cross-sectional study was undertaken from November 1 –30, 2020. A multi-stage sampling technique was employed and a total of 1083 adolescents (361 from the urban and 722 from the rural areas) were selected randomly to take part in the study. Pre-tested, interviewer-administered, structured questionnaires were used to collect the data. The data were encoded and entered into Epi-Data version 3.1 and then exported to SPSS version 23 for analysis. χ2 test was computed to see a significant difference in SRH service utilization among urban and rural adolescents. In a bivariable logistic regression analysis, a variable with a p-value less than 0.25 has been selected for a multivariable logistic regression model. Variables with p-values less than 0.05 were declared statistically significant in multivariate logistic regression.

**Results:**

A total of 1,075 adolescents (358 from urban and 717 from rural) took part in the study, yielding a response rate of 99.3%. The overall SRH service utilization among the whole adolescents was 39.5% (95%CI: 36.5, 42.4). There was a significant difference in SRH service utilization between urban 56.9% (95%CI: 51.8, 62.1) and rural 30.8% (95%CI: 27.4, 34.2) adolescents (χ2 = 68.3, *p* < 0.001). Residence[AOR = 2.62; 95%CI:1.63,3.41], availability of youth clubs [AOR = 4.73; 95%CI:3.43,6.53], taking part in peer education [AOR = 2.06; 95%CI:1.48,3.88], having parental discussion [AOR = 3.29; 95%CI:1.73,3.33], and being knowledgeable on SRH issues [AOR = 2.01; 95%CI: 1.45,3.03] were identified as a significant determinants of SRH service uptake. Having parental discussion, geographical accessibility, and knowledge on SRH were significant predictors of SRH service uptake among rural adolescents.

**Conclusion:**

Overall, ASRH service utilization in the study area was low, despite urban adolescent service uptake becoming higher than rural adolescents. Since the majority of adolescents were enrolled in schools, schools should be an area of intervention to improve adolescents' knowledge of SRH services through mass media, community networks, and interpersonal/group communication. Furthermore, promoting parent-adolescent discussions, as well as peer-to-peer discussions at the family and school level, should be emphasized. Stakeholders in the education and health sectors need to strengthen their efforts to establish youth clubs in places where they do not yet exist, especially in rural schools.

**Supplementary Information:**

The online version contains supplementary material available at 10.1186/s12889-022-12634-x.

## Background

Adolescence is a crucial period of human development, characterized by rapid physical, psychosocial, intellectual, and emotional maturation, as well as erotic and reproductive maturation [1, 2]. Healthy adolescents contribute to economic development by rising productivity and preventing the spread of disease through generations. Every dollar spent on adolescent health returns tenfold in terms of health, social, and economic benefits [3].

Adolescent sexual and reproductive health (ASRH) services are described as a set of strategies, procedures, and services aimed at preventing and treating sexual health problems in adolescents while also promoting their overall well-being [4, 5]. It encourages adolescents' physical and emotional well-being by addressing their desire to avoid unintended pregnancy, unsafe abortion, sexually transmitted infections (STIs) (including HIV/AIDS), and other forms of sexual harassment and pressure [6, 7]. The constellation of the ASRH services are; provision of information and education on SRH issues, counseling, and provision of modern contraception, volunteered HIV/AIDS counseling, and testing(VCT), STI diagnosis and management, and safe and/or post-abortion care [6–8].

Adolescents number up to 1.2 billion worldwide, with 513 million between the ages of 15 and 19, and 85 percent of them live in developing countries [9]. They account for up to a quarter of the population in some countries, and their numbers are expected to rise by 2050, especially in low- and middle-income countries (LMICs), where access to health and social services, employment, and livelihoods appears to be under strain [10–12]. In sub-Saharan Africa, adolescents make up 23% of the population of the region [11]. Around 25% of Ethiopia's total population is covered by a cohort of adolescents [6, 13].

While the Convention on the Rights of the Child (CRC) protects adolescents' right to SHR services, neither the providers of these services nor the systems under which they operate are equipped to recognize adolescents' needs and benefits [14–16]. In developing countries such as Ethiopia, health systems and programs are primarily designed for young children or adults, with SRH services for adolescents, particularly those living in rural areas, having received less attention [15]. Although under-five mortality decreased by half during the duration of the Millennium Development Goals [17], advances in adolescent mortality have been delayed [16]. There are over 1.2 million adolescent deaths worldwide every year [10, 16].

Neglecting adolescent SRH services has serious consequences; adolescent girls, in particular, are at higher risk of unintended pregnancy, HIV and sexually transmitted infections (STIs), sexual coercion, exploitation, and violence [12, 18]. Every year, nearly 16 million adolescent girls give birth, with the majority of these births occurring in the context of early marriage, and 90% of these births occurring in developing countries [19]. 7.4 million teen girls have become pregnant unintentionally, due to a shortage of contraceptive options [20]. Up to 68% of adolescents in sub-Saharan Africa have an unmet need for contraception [20]. In Ethiopia, a third of girls aged 15 to 19 have started having children, with rural settings dominant [21].

According to the 2019 Mini Ethiopian Demographic and Health Survey (mini-EDHS 2019), contraceptive usage among currently married women aged 15–19 years was just 36.5%, with injectable, implants, and IUD use of 27.5%, 5.9%, and 0.0%, correspondingly [22]. With 75% and 80% respectively, the proportion of adolescents who have never been tested for HIV is highest among women and men aged 15–19 [21]. Limited studies conducted elsewhere in Ethiopia have shown that inadequate access to SRH services in general with a range of 21.5–41.2% [23–25].

The Federal Ministry of Health (FMOH) of Ethiopia has introduced various strategies to facilitate national-level adolescent and youth reproductive health services to overcome SRH problems [6, 13]. Despite these efforts, Ethiopian adolescents and young adults continue to face significant challenges to reproductive health services [6]. Adolescents in rural and urban areas have different socio-demographic, socioeconomic, and cultural characteristics, which influence how they are using SRH services.

Although studies on the uptake of ASRH services in Ethiopia have been conducted [23–25], they have failed to show the disparity in service uptake among rural and urban settings. Once the extent and determinants of ASRH service uptake in urban and rural contexts are known, it will be crucial to provide evidence-based information and recommendations for potential interventions to reduce the burden of disease and disability among adolescents. Furthermore, identifying and recognizing the pattern of SRH service utilization among adolescents can aid in future planning for better service delivery. Hence, this study aimed at assessing the level and determinants of ASRH service utilization among adolescents living in urban and rural districts of the Guraghe zone, southern Ethiopia.

## Methods

### Study setting, period, and design

From November 1 to 30, 2020, a community-based comparative cross-sectional study has been undertaken in the Guraghe Zone, Southern Ethiopia. The zone is 158 km from Ethiopia's capital, Addis Ababa, and 337 km from Hawassa (the capital city of southern nation nationalities and people region). For the fiscal year 2020, the Zone's total population was 1,807,689, with 1,468,940 (81.3%) and 338,749 (18.7%) living in rural and urban districts, respectively. Adolescents between the ages of 15 and 19 constituted 13.6% (245,845) of the total population. The zone is divided into nine rural districts and four urban administrative, comprised of 174 rural and 27 urban kebeles (*the smallest administrative unit next to a district in the Ethiopian government*). There are 128 health facilities which are quantified as 74 health centers, 5 hospitals, 168 health posts, 30 private clinics.

### The population of the study

The source populations were all adolescents of age 15–19 years living in the urban and rural districts of Guraghe zone, whereas the study population consisted of all selected adolescents living in the selected districts during the study period. Adolescents who have lived in the study area for less than six months and those who were seriously ill at the time of data collection were excluded from the study.

### Sample size determination

The sample size was determined by applying a double population proportion formula via StatCalc menu of Epi Info version7.1; considering the proportion of SRH service uptake in Urban = 33.8% [24] and proportion of SRH service uptake in rural = 21.5% [23], a 5% margin of error, power of 80%, 1:2 urban to rural ratio. Based on the above assumptions, the sample size was 492 (164 from urban and 328 from rural), and after allowing for a 10% nonresponse rate, and design effect of 2, the final sample size for this study was 1083(361 from urban and 722 rural).

### Sampling procedure

**Fig. 1:**
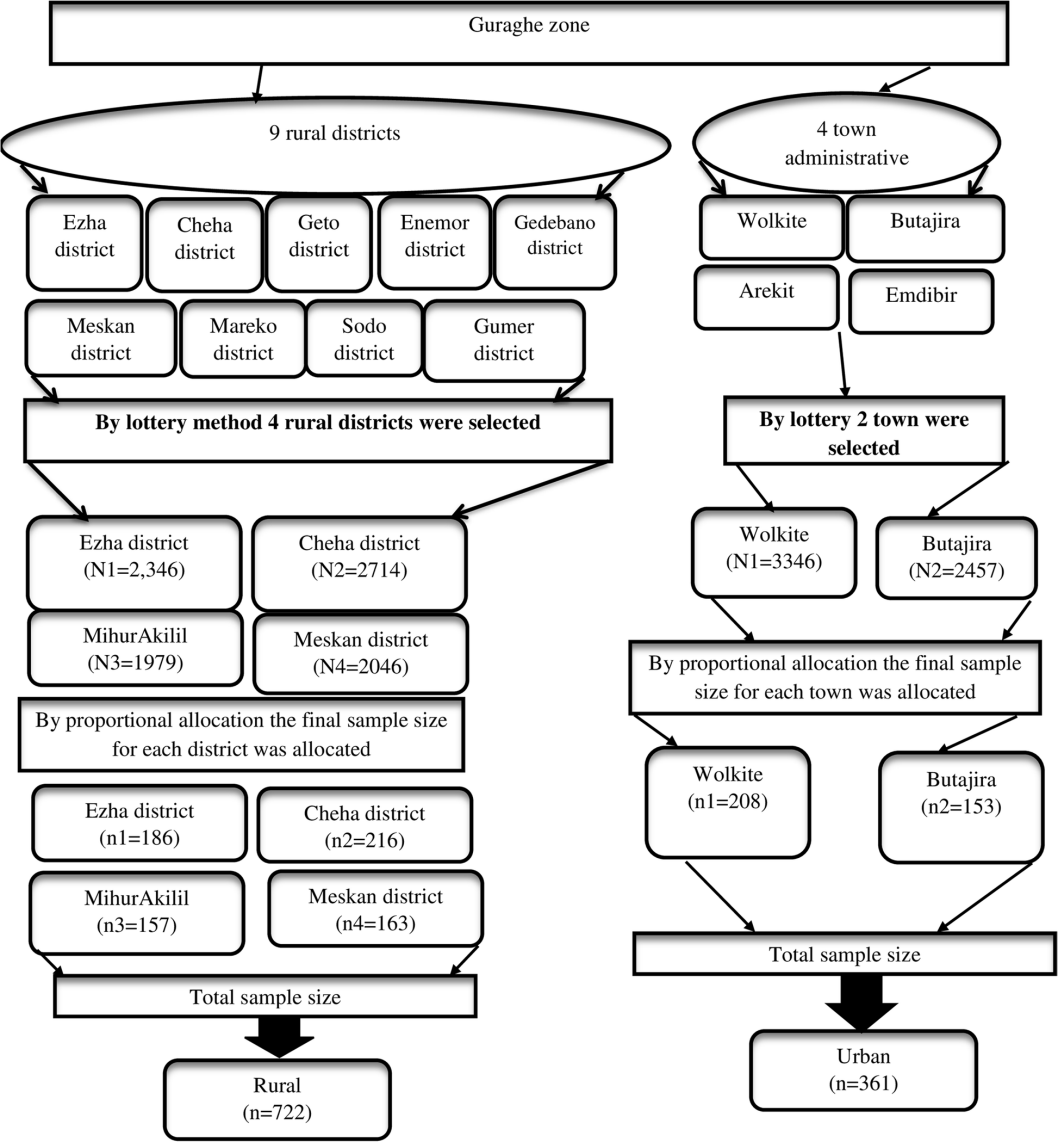
Schematic presentation of sampling procedures followed to get study participants in Guraghe zone, southern Ethiopia, 2020

A multi-stage sampling technique was used to access study participants. Initially, the zone was divided into rural districts and urban administrative. In the first stage, the lottery method was used to select four of the ten rural districts and two of the four town administrative (urban). The two selected town administrative (Urban) were Wolkite town (with 8kebeles) and Butajira town (with 9 kebeles). At stage two 20 rural kebeles, were randomly selected from a total of 59 kebeles in the four rural districts mentioned above. Of the 17 urban kebeles, 7 were selected by lottery method. Finally, the sample size was proportionally allocated to each selected kebele (Fig. 1). With the assistance of community health workers (CHWs), households with eligible participants were coded and a sampling frame was set up. It was practical to access each study participant by simple random sampling (i.e. computer-generated random number). When there was more than one deserving adolescent in the selected household, a lottery method was used.

### Data collection tools, methods, and personnel

After reviewing previously conducted related studies in the areas of interest, pre-tested structured questionnaires have been developed [13, 15, 23–25]. The questionnaire had several sections, including socioeconomic and demographic characteristics, access to SRH services, knowledge of SRH-related issues, respondents' lifestyle and sexual activity, and utilization of RH services. The data was collected by14 diploma nurses with prior data collection expertise under the supervision of six public health officers via a face-to-face interview. All data collectors and supervisors got a one-day intensive training on the study's purpose, methodologies, and data collection techniques. The interviewers returned to the families at least three times at different time intervals when the eligible study participants were not present during data collection. After three visits, if the interviewer was still unable to contact the study participants, he or she went on to the next allocated household.

### Data quality management

The questionnaire was written in English first, then translated into the local language by fluent speakers, and then retranslated back into English by another translator to assure consistency. One week before the actual data collection, a pre-test was conducted on 54 adolescents (5 percent of the sample total) in one of the unselected districts. Based on the results of the pre-test, all necessary changes were made to improve the completion of the questionnaires. Confusing and long questions were removed and shortened as a result of the pre-test.The principal investigator and field supervisors closely oversaw and coordinated the total data collection process. Before analysis, all of the data were double-checked for completeness and consistency. The data were taken in a quiet area of the study participant's home where there was no noise or disturbance.

### Definition and operationalization of study variables

Sexual and reproductive health (SRH) service utilization: when adolescents received at least one of the five important elements of SRH services; information and education on SRH matters, consultation and provision of modern contraceptives; STIs diagnosis and management, getting VCT service, and abortion and/or post-abortion care within the last 12 months [24, 25].

Adolescent: In this study, adolescents denote boys and girls between the ages of 15–19 [24, 26, 27].

Parental Discussion on SRH issues: Adolescents who have discussed at least two SRH topics in the previous 12 months(Condom use, STI/HIV/AIDS, abstinence, unwanted pregnancy, contraception) together with their parent/s [24].

Sexual exposure history: Adolescents who had sex in their lives have been identified as having a sexual encounter history and not otherwise [24]

Modern contraceptive service utilization: Adolescents that during the past 12 months have used any of the modern methods of birth control (oral contraceptives, condoms (male and female), injectables, implants, intrauterine devices, emergency contraceptive pills, and spermicidal agents) [24, 26].

Accessibility to SRH service: Applied to the perceived distance traveled by respondents to reach SRH service delivery points and/or time spent by them. Adolescents residing within a 1.6-km radius of the nearest SRH service center and/or reaching those service delivery points within a walking distance of fewer than 30 min were graded as having good and otherwise poor geographical mobility[24].

Substance use: Using addictive substances such as alcohol, ‘khat’, or cigarettes with either frequency of; more repeated than daily, daily, weekly or monthly in the past 12 months before the study [24].

Reproductive health service knowledge: Twelve questions were asked to adolescents encompassing the perceptions about SRH issues. An index that summarizes the level of knowledge and categorizes it as Knowledgeable if the summary index is equal to or greater than the mean [23, 24].

Availability of Youth clubs: Accessibility of places/rooms where young people can meet and gather SRH information, SRH services such as contraceptives, physical activities, social support, peer-to-peer discussion, with the aid of trained workers and volunteers to protect adolescents from negative events, anti-social behavior, crime, drug, and alcohol abuse that are a problem in this community [28–30].

### Data analysis

The data entry was done using EPI Data 3.1 and exported for analysis to SPSS version 23. Using descriptive statistical analysis, frequency, percentage, and mean for explanatory and response variables were run. Chi-square testing was done to see if there was any significant on SRH service uptake among urban and rural adolescents and a statistically significant difference was observed between the two groups (χ2 = 68.3, *p* < 0.001), indicating that the factors associated with SRH utilization could be different among rural and urban groups. Therefore, the analysis was conducted separately. Bivariable logistic regression was used to find out the relationship between SRH service utilization and the independent variables, and variables with a p-value of less than 0.25 were selected candidates for a multivariable logistic regression(MLR) model. Variables with a p-value less than 0.05 were considered statistically significant in multivariable logistic regression analysis. The AOR and its 95%CI were used to report the association between SRH service utilization and explanatory variables. The model fitness was assessed using the Hosmer and Lemeshow goodness of fit tests, which yielded a score of 0.59. The variance inflation factors (VIF > 10) were used to check for multicollinearity amongst the explanatory variables.

## Results

### Socio-demographic characteristics of respondents

A total of 1,075 adolescents (358 from urban and 717 from rural) took part in the study, yielding a response rate of 99.3%. The mean (± SD) age of the adolescents was 17.0 ± 1.4 years. Females make up a majority of respondents (622, or 57.9%), with 57.3% and 59.7% living in urban and rural areas, respectively. Over half of all respondents, 560(52.1%), attended high school, with 196 (54.7%) in an urban and 364 (50.8%) in a rural setting. The majority of adolescents in both groups belong to the Guraghe ethnic group (91.6% in the urban and 91.4% in the rural) and followers of orthodox religion (urban:56.4.0% and rural:53.8%) (Table 1).

**Table 1:** Socio-demographic characteristics of rural and urban adolescents in Guraghe zone, Southern Ethiopia, 2020

Variable categories	Urban (*n* = 358)	Rural(*n* = 717)	Total = 1075	Test statistics
	**n(%)**	**n(%)**	**n(%)**	
**Age (*n* = 1075)**				
15–16	152(42.4)	299(41.7)	451(41.9)	χ2 = 0.056 *P* = 0.432
17–19	206(57.6)	418(58.3)	624(58.1)	
Mean(± SD) age (*n* = 1075)	16.9 ± 1.4	17.0 ± 1.4		
**Sex (*n* = 1075)**				
Male	153(42.7)	289(40.3)	442(41.1)	
Female	205(57.3)	428(59.7)	633(58.9)	
**Marital status(*n* = 1075)**				
Ever Married	22(6.1)	34(4.7)	56(5.2)	χ2 = 0.952 *P* = 0.202
Unmarried	336(93.9)	683(95.3)	1019(94.8)	
**Educational status (*n* = 1075)**				
No formal education	12(3.4)	30(4.2)	42(3.9)	χ2 = 1.688 *P* = 0.430
Primary	150(41.9)	323(45.0)	473(44.0)	
Secondary	196(54.7)	364(50.8)	560(52.1)	
**Occupational status (*n* = 1075)**				
Student	283(79.1)	590(82.3)	873(81.2)	χ2 = 2.030 *P* = 0.362
Daily laborer	41(11.4)	75(10.4)	116(10.8)	
Unemployed	34(9.5)	52(7.3)	86(8.0)	
**Current living arrangement (*n* = 1075)**				
With bother parent	301(84.1)	598(83.4)	899(83.6)	χ2 = 5.62 *P* = 0.132
With mother only	23(6.4)	61(8.5)	84(7.8)	
With father only	20(5.6)	24(3.4)	44(4.1)	
With husband or wife	14(3.9)	34(4.7)	48(4.5)	
**Mother’s education level (*n* = 1032)**				
No Formal education	153(45.1)	398(57.4)	551(53.4)	χ2 = 26.904 *P* < 0.001
Primary	123(36.3)	198(28.6)	321(31.1)	
Secondary	29(8.6)	71(10.2)	100(9.7)	
Diploma and above	34(10.0)	26(3.8)	60(5.8)	
**Father’s educational level (*n* = 988)**				
No formal education	86(25.9)	252(38.4)	338(34.2)	χ2 = 62.872 *P* < 0.001
Primary	83(25.0)	243(37.0)	326(33.0)	
Secondary	109(32.8)	120(18.3)	229(27.2)	
Diploma and above	54(16.3)	41(6.3)	95(9.6)	
**Family size(*n* = 1075)**				
≤ 5	223(62.3)	349(48.7)	572(53.2)	χ2 = 18.876 *P* < 0.001
> 5	135(37.7)	368(51.3)	503(46.8)	

### Geographical accessibility to ASRH service delivery points

**Fig. 2:**
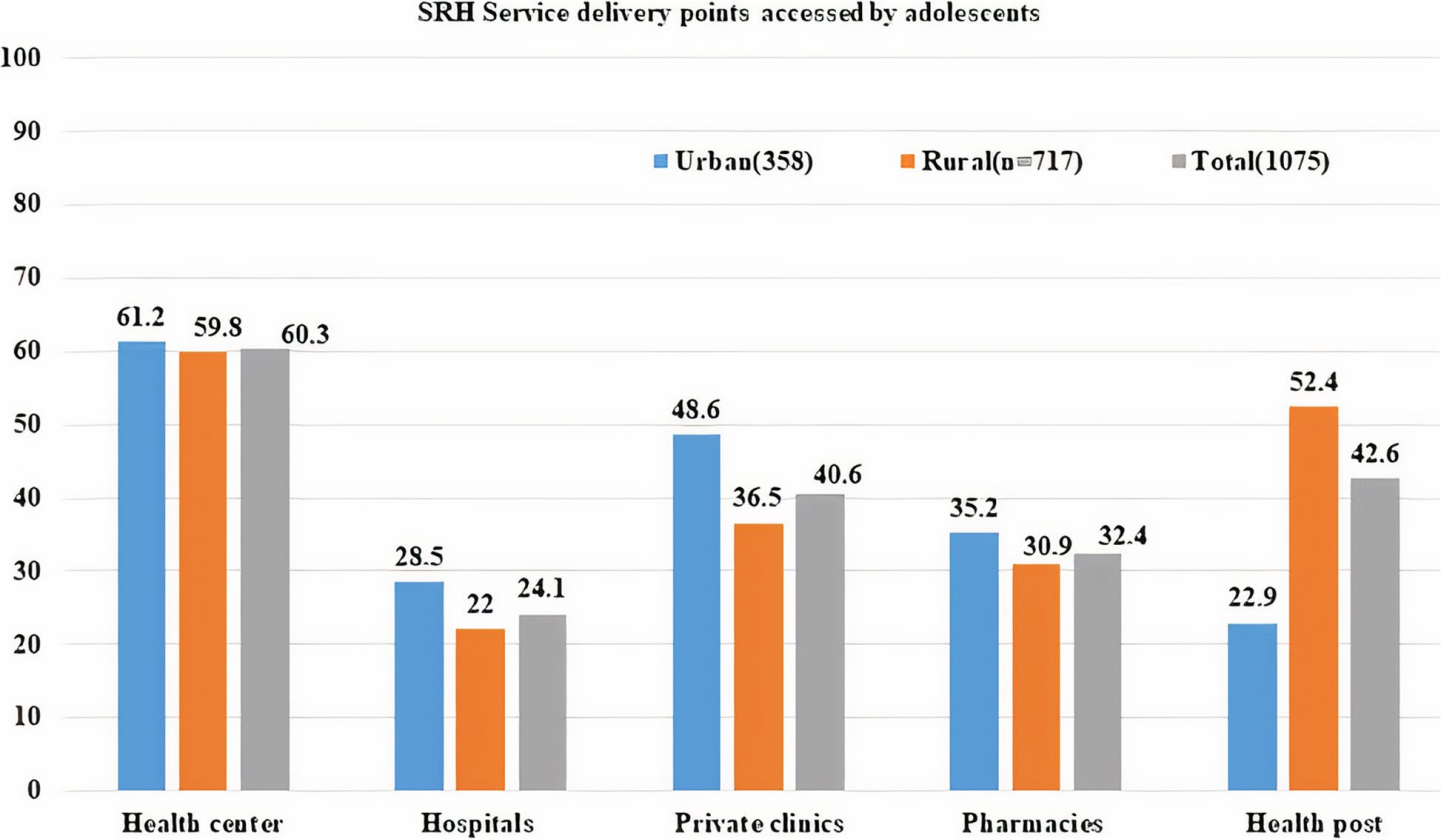
Service delivery points accessed by adolescents for SRH service uptake in Guraghe zone, southern Ethiopia, 2020

In terms of geographic accessibility, 264 (73.7%) of urban adolescents and 183 (25.5%) of rural adolescents were able to access SRH service delivery points within a 30-min walk of their home (χ2 = 228.18, *p* < 0.001). Health centers and private clinics were among the service delivery points frequently accessed by 219 (61.2%) and 174 (48.5%) of urban adolescents, respectively (Fig. 2). A significant difference in the availability of youth clubs in urban and rural settings in which more than half, 192(53.6%) of rural and 268(37.4%) urban adolescents reported that availability of youth clubs (YCs) in their nearby environment (χ2 = 25.767, *p* < 0.001).

### Adolescents’ attributes related to sexual and reproductive health

**Fig. 3:**
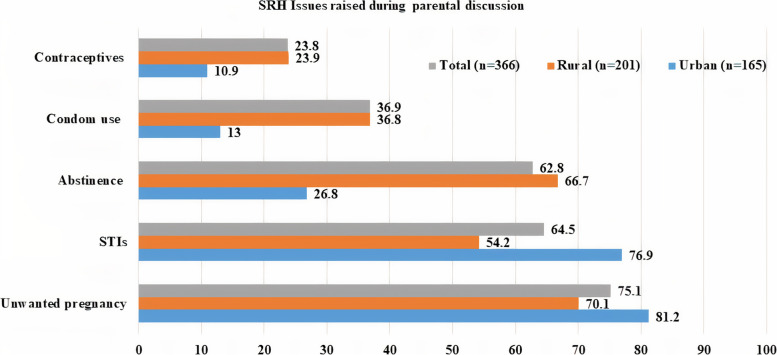
List of SRH issues raised during a parental discussion among adolescents of Guraghe zone, Southern Ethiopia, 2020

A total of 227 (21.1%) adolescents reported having had a sexual partner/s in their lifetime, with 115 (urban: 57 (62.6%) and rural: 58 (42.6%) having had sexual intercourse at least once. Between urban and rural adolescents, there was a significant difference in having a parental discussion in the previous 12 months(urban = 165(46.1%); Rural = 208(29.1%), χ2 = 30.741, *P* < 0.001). Unwanted pregnancy was the most common topic addressed during a parental discussion among both urban and rural adolescents, with 134 (81.2%) and 141 (70.1%), respectively (Fig. 3). In terms of substance use, 91 (25.4%), 106 (29.5%), and 46 (12.8%) of urban adolescents drunk alcohol chew ‘Khat,' and smoke cigarettes, respectively (Table 2).

**Table 2:** Individual attributes related to sexuality and reproductive health among rural adolescents of Guraghe zone, southern Ethiopia, 2020

Variable categories	Urban = 358	Rural = 717	Total = 1075	Test statistics
	**n(%)**	**n(%)**	**n(%)**	
**Ever Had sexual partner/s(*n* = 1075)**				
Yes	91(25.4)	136(18.9)	227(21.1)	χ2 = 5.966 *P* = 0.015
No	267(74.6)	581(81.1)	848(78.9)	
**Number of sexual partners(*n* = 227)**				
One	55(60.4)	109(80.1)	164(72.2)	χ2 = 2.887 *P* = 0.1342
Two	27(29.7)	25(18.4)	52(22.9)	
More than two	9(9.9)	2(1.5)	11(4.9)	
**Ever had sexual intercourse (*n* = 227)**				
Yes	57(62.6)	58(42.6)	115(50.7)	χ2 = 6.755 *P* = 0.011
No	34(37.4)	78(57.4)	112(49.3)	
**Ever consumed Alcohol(*n* = 1075)**				
Yes	91(25.4)	151(21.1)	242(22.5)	χ2 = 2.601 *P* = 0.063
No	267(74.6)	566(78.9)	833(77.5)	
**Frequency of alcohol consumption (*n* = 242)**				
Almost every day	4(4.4)	11(7.3)	15(6.2)	
At least once a week	8(8.8)	14(9.3)	22(9.1)	
At least once a month	13(14.3)	16(10.6)	29(12.0)	
At least once a year	56(61.5)	87(57.6)	143(59.1)	
Ceased currently^a^	10(11.0)	23(15.2)	33(13.6)	
**He/she Ever chew ‘Khat’ (*n* = 1075)**				
Yes	106(29.6)	178(24.8)	284(26.4)	χ2 = 2.810 *P* = 0.055
No	252(70.4)	539(75.2)	791(73.6)	
**Frequency khat chewing(*n* = 284)**				
Almost every day	6(5.7)	2(1.1)	8(2.8)	
At least once a week	37(34.9)	68(38.2)	105(37.0)	
At least once a month	41(38.7)	74(41.6)	115(40.5)	
At least once a year	10(9.4)	14(7.9)	24(8.4)	
Not current users^a^	12(11.3)	20(11.2)	32(11.3)	
**She/he ever smoke a cigarette (*n* = 1075)**				
Yes	46(12.8)	63(8.8)	109(10.1)	χ2 = 4.262 *P* = 0.026
No	312(87.2)	654(91.2)	966(89.9)	
**Frequency smoking(*n* = 109)**				
Almost every day	2(4.3)	3(4.8)	5(4.6)	
At least once a week	6(13.0)	4(6.3)	10(9.2)	
At least once a month	9(19.6)	8(12.7)	17(15.6)	
At least once a year	9(19.6)	24(38.1)	33(30.3)	
Ceased currently^a^	20(43.5)	24(38.1)	44(40.4)	

^a^Adolescents who have taken none of the above substances in the past three months

### Knowledge of adolescents about SRH related issues

The level of knowledge of adolescents on SRH issues was assessed using eleven items. As a result, study participants had a mean (± SD) knowledge score of 5.4 ± 2.4 (5.7 ± 2.7 in urban and 5.2 ± 2.3 in rural). Nearly half of the study participants, 508 (47.2%), had a good knowledge of SRH issues, with 186 (51.9%) in the urban and 322 (44.9%) in the rural. Nearly seven out of ten (69.3%) of urban and more than half (56.8%) of rural adolescents had information about SRH services and the school environment was found to be the most popular source of information among both urban and rural. At least one form of SRH service that should be provided to an adolescent is reported by 221 (61.7%) of urban and 343 (47.3%) of rural adolescents (Table 3).

**Table 3:** Level of SRH knowledge of adolescents of Guraghe zone, Southern Ethiopia, 2020

Variable categories	Urban = 358	Rural = 717	Total = 1075	χ2	P-value
	**n(%)**	**n(%)**	**n(%)**		
**Ever heard about SRH (*n* = 1075)**					
Yes	257(71.8)	353(49.2)	610(56.7)	49.489	< 0.001
No	101(28.2)	364(50.8)	465(43.3)		
**Source of information(*n* = 610)**					
From school	256(99.6)	280(79.3)	536(87.9)	57.443	< 0.001
Radio	186(72.4)	205(58.1)	351(57.5)	13.215	< 0.001
Television	116(45.3)	67(18.9)	183(30.0)	48.451	< 0.001
Family members	52(20.2)	133(36.7)	185(30.3)	22.609	< 0.001
Social media	45(17.5)	55(15.6)	100(16.4)	0.691	0.231
**Can mention at least one SRH service that should be delivered to an adolescent(*n* = 1075)**					
Yes	230(64.2)	361(50.3)	591(55.0)	18.631	< 0.001
No	128(35.8)	356(49.7)	484(45.0)		
**Know SRH service delivery points (*n* = 1075)**					
Yes	206(57.6)	360(50.2)	566(52.7)	5.150	0.014
No	152(42.4)	357(49.8)	509(47.3)		
**Know SRH service provider(*n* = 1075)**					
Yes	229(63.9)	323(45.1)	552(51.3)	34.206	< 0.001
No	129(36.1)	394(54.9)	523(48.7)		
**Know the reasons for unintended pregnancy**					
Yes	229(63.9)	431(60.1)	660(61.4)	1.497	0.123
No	129(36.1)	286(39.9)	415(38.6)		
**Know at least one way of avoiding pregnancy**					
Yes	190(53.1)	412(57.5)	602(56.0)	1.867	0.097
No	168(46.9)	305(42.5)	473(44.0)		
**Know at least one type of STI**					
Yes	213(59.5)	383(53.4)	596(55.4)	3.573	0.034
No	145(40.5)	334(46.6)	479(44.6)		
**Know at least one method of STI prevention**					
Yes	201(56.1)	419(58.4)	620(57.7)	0.514	0.257
No	157(43.9)	298(41.6)	455(42.3)		
**Know the place where STI cases are managed**					
Yes	137(38.3)	261(36.4)	398(37.0)	0.357	0.297
No	221(61.7)	456(63.6)	677(63.0)		
**Know at least one benefits of contraceptives**					
Yes	248(69.3)	479(66.8)	727(67.6)	0.664	0.228
No	110(30.7)	238(33.2)	348(32.4)		
**Know at least one type of contraceptive**					
Yes	222(62.0)	431(60.1)	653(60.7)	0.361	0.297
No	136(38.0)	286(39.9)	422(39.3)		
**Overall knowledge**					
Knowledgeable	186(51.9)	322(44.9)	508(47.2)	4.756	0.029
Not knowledgeable	172(48.1)	395(55.1)	567(52.8)		

### Uptake of SRH services among adolescents

**Fig. 4:**
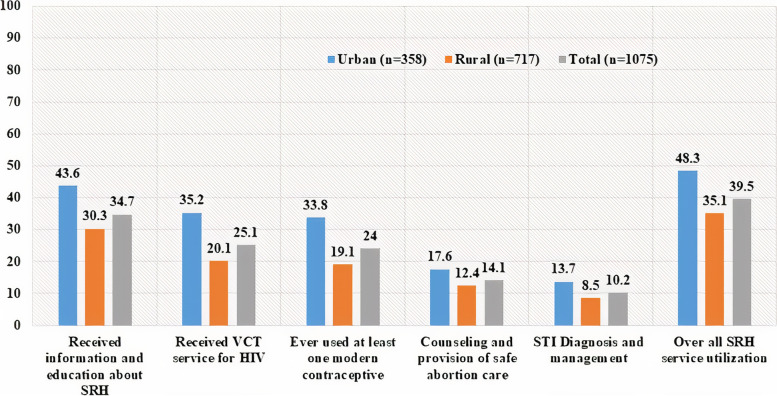
Shows distribution SRH services utilization by service item among adolescents in Guraghe zone, southern Ethiopia, 2020

The overall utilization of SRH service by adolescents was 39.5% (95%CI: 36.5, 42.4). There was a significant difference in the rate of SRH service utilization between urban 204(56.9%) (95%CI: 51.8, 62.1) and rural 221(30.8%) (95%CI: 27.4, 34.2) adolescents (χ2 = 68.3, *p* < 0.001). SRH information and education was the most frequent SRH service item received by both urban and rural adolescents, with 156 (43.6%) and 217 (30.3%) respectively. Only 13.7% of urban and 8.5% of rural respondents received STI Diagnosis and Management services (Fig. 4).

### Reasons for not using SRH service among adolescents

**Fig. 5:**
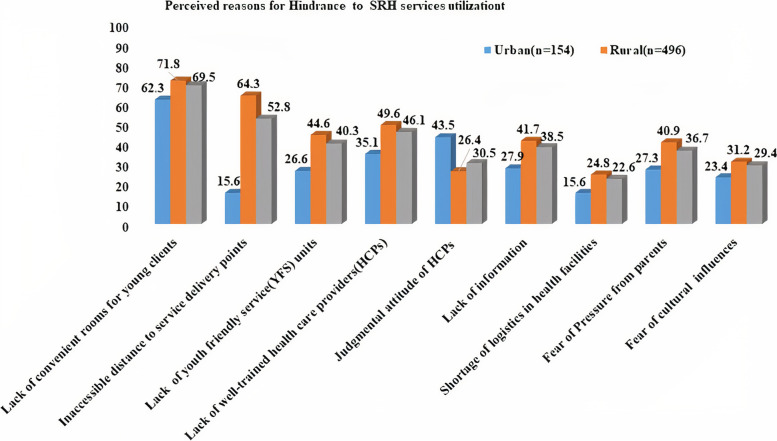
Reasons mentioned by adolescents as a barrier for not using SRH services in Guraghe zone, Southern Ethiopia, 2020

Lack of convenient or separate rooms for SRH service delivery was reported by 452 (69.5%) adolescents as the most prevalent hindrance to SRH service uptake (urban: 96 (62.3%) and rural: 356(71.8%). Lack of well-trained health care providers(HCPs) on SRH service was the other reason mentioned by 54(35.1%) of urban adolescents. Far distance to nearby health facilities was another prominent reason mentioned by 319(64.3%) of rural adolescents (Fig. 5).

### Determinants of SRH service utilization

To identify determinants of SRH service uptake, we fitted three different models. The first model was used to assess the overall factors that determine the use of SRH services. Five variables were identified as significant determinants of SRH service uptake among the entire adolescents in MLR analysis: residence, having a peer-to-peer education, having a parental discussion, availability of YFS units, and being knowledgeable on SRH issues.

Urban adolescents had a 2.64 times greater chance of accessing SRH services than rural adolescents [AOR = 2.64; 95%CI: 1.63, 3.41]. Adolescents who took part in peer-to-peer education were twice as likely as their counterparts to use SRH services [AOR = 2.06; 95%CI: 1.48, 3.88]. The presence of Youth clubs in their immediate vicinity influences the use of SRH services. Adolescents who lived in areas with functioning youth clubs(YCs) units had a 4.7 times greater chance of using SRH services than their counterparts [AOR = 4.73; 95% CI:3.43,6.53]. It was revealed that having a parent discussion about SRH had a positive influence on SRH service adoption. Adolescents who had a parental discussion had a 3.3 times higher chance of using SRH services than those who did not [AOR = 3.29; 95% CI: 2.36, 5.59]. Furthermore, adolescents who were knowledgeable on SRH matters had a 2 times higher likelihood of using SRH services than their counterparts [AOR = 2.01; 95% CI: 1.45, 3.03] (Table 4).

**Table 4:** Determinants of SRH Service Utilization among urban and rural adolescents in Guraghe Zone, Southern Ethiopia, 2020

Variable categories	SRH services utilization				
	Yes (%)	No (%)	COR(95%CI)	AOR(95%CI)	p-value
**Residence**					
Rural	221(52.0)	496(76.3)	1	1	1
Urban	204(48.0)	154(23.7)	2.97(2.29,3.86)^a^	2.64(1.63,3.41)^b^	< 0.001
**Age( in years)**					
15–16	171(40.2)	280(43.1)	1		
17–19	254(59.8)	370(56.9)	1.12(0.88,1.44)		
**Sex**					
Female	234(55.1)	399(61.4)	1	1	
Male	191(44.9)	251(38.6)	1.29(1.01,1.66)^a^	1.26(0.91,1.75)	0.158
**Current school enrolment**					
No	31(7.3)	68(10.5)	1		
Yes	394(92.7)	582(89.5)	1.48(0.95,2.31)^a^	1.26(0.69,2.30)	0.460
**Educational status**					
No formal education	12(2.8)	30(4.6)	1	1	
Primary	155(36.5)	318(48.9)	1.22(0.61,2.44)	0.87(0.34,2.23)	0.769
Secondary	258(60.7)	302(46.5)	2.14(1.07,4.26)^a^	1.29(0.50,3.28)	0.600
**Current living arrangement**					
With husband/ wife	13(3.1)	35(5.4)	1		
With father only	16(3.8)	28(4.3)	1.54(0.63,3.73)		
With mother only	31(7.3)	53(8.2)	1.57(0.72,3.42)		
With bother parent	365(85.9)	534(82.1)	1.64(0.86,3.53)		
**Mother’s education level**					
No Formal education	215(52.7)	336(53.9)	1		1
Primary	108(26.4)	213(34.1)	0.79(0.59,1.06)	0.68(0.47,0.98)	0.071
Secondary	48(11.8)	52(8.3)	1.44(0.94,2.21)^a^	1.48(0.87,2.52)	0.152
Diploma and above	37(9.1)	23(3.7)	2.51(1.45,4.35)^a^	1.72(0.86,3.45)	0.127
**Father’s educational level**					
No formal education	126(32.3)	212(35.5)	1		1
Primary	112(28.6)	214(35.8)	0.88(0.64,1.21)	1.13(0.75,1.68)	0.558
Secondary	101(25.8)	128(21.5)	1.33(0.94,1.87)^a^	1.11(0.72,1.71)	0.622
Diploma and above	52(13.3)	43(7.2)	2.03(1.28,3.22)^a^	1.33(0.73,2.39)	0.350
**Family size**					
> 5	195(45.9)	308(47.4)	1		
≤ 5	230(54.1)	342(52.6)	1.06(0.83,1.36)		
**Geographical accessibility**					
Far(≥ 30 min)	207(48.7)	421(64.8)	1	1	
Close (< 30 min)	218(51.3)	229(35.2)	1.94(1.51,2.48)^a^	1.04(0.73,1.49)	0.813
**Availability of youth clubs**					
No	144(33.9)	471(72.5)	1		
Yes	281(66.1)	179(27.5)	5.13(3.94,6.69)^a^	4.73(3.43,6.53)^b^	< 0.001
**Ever had sexual partner/s**					
No	326(76.7)	522(80.3)	1	1	
Yes	99(23.3)	128(19.7)	1.24(0.92,1.67)^a^	1.44(0.98,2.13)	0.065
**Had a parental discussion on SRH issues**					
No	190(44.7)	512(78.8)	1	1	1
Yes	235(55.3)	138(21.2)	4.58(3.54,6.06)^a^	3.29(2.36,5.59)^b^	0.001
**Participated in peer to peer education**					
No	120(28.2)	320(49.2)	1		
Yes	305(71.8)	330(50.8)	2.46(1.89,3.20)^a^	2.06(1.47,3.88)	0.015
**Knowledge of SRH issues**					
Not knowledgeable	165(38.8)	402(61.9)	1	1	
Knowledgeable	260(61.2)	248(38.1)	2.55(1.99,3.28)^a^	2.01(1.45,3.03)	0.001

Key: 1: Reference category; *AOR* Adjusted odds ratio, *COR* Crude odds ratio

^a^statistically significant at *p*-value < 0.25, ^b^ statistically significant at *p*-value < 0.05

The second model was fitted only for urban adolescents, and two variables were identified as significant determinants of SRH service uptake: the availability of youth clubs in their local environment and having a parental discussion about SRH issues. Adolescents who lived in areas with functioning youth clubs were 5 times more likely to use SRH services than their counterparts[AOR = 5.06; 95%CI: 2.92,8.77]. Adolescents who had a parental discussion about SRH topics within the last 12 months had a more than 3 times greater chance of receiving SRH services than those who did not [AOR = 3.25; 95% CI: 2.01,5.67] (Table 5).

**Table 5:** Determinants of SRH Service Utilization among urban adolescents in Guraghe Zone, Southern Ethiopia, 2020

Variable categories	SRH services utilization		COR(95%CI)	AOR(95%CI)	p-value
	Yes (%)	No (%)			
**Age( in years)**					
15–16	82(40.2)	70(45.4)	1		
17–19	122(59.8)	84(54.6)	1.24(0.81,1.89)		
**Sex**					
Female	111(54.4)	94(61.0)	1	1	
Male	93(45.6)	60(39.0)	1.31(0.86,2.01)^a^	1.31(0.75,2.29)	0.342
**Current enrolment at school**					
No	16(7.8)	21(13.6)	1	1	
Yes	188(92.2)	133(86.4)	1.86(0.93,3.68)^a^	1.74(0.70,4.28)	0.230
**Educational status**					
No formal education	6(2.9)	6(3.9)	1	1	
Primary	66(32.4)	84(54.5)	0.79(0.24,2.55)	0.65(0.15,2.93)	0.578
Secondary	132(64.7)	64(41.6)	2.06(0.64,6.65)^a^	1.30(0.29,5.83)	0.727
**Current living arrangement**					
With husband/ wife	8(3.9)	6(3.9)	1		
With father only	9(4.4)	11(7.2)	0.61(0.15,2.43)		
With mother only	14(6.9)	9(5.8)	1.17(0.30,4.49)		
With bother parent	173(84.8)	128(83.1)	1.01(0.34,2.99)		
**Mother’s education level**					
No Formal education	78(40.2)	75(51.7)	1	1	1
Primary	72(37.1)	51(35.2)	1.36(0.84,2.19)^a^	1.37(0.49,3.76)	0.541
Secondary	20(10.3)	9(6.2)	2.14(0.91,4.99)^a^	1.82(0.64,5.18)	0.258
Diploma and above	24(12.4)	10(6.9)	2.31(1.03,5.15)^a^	1.63(0.87,3.03)	0.126
**Father’s educational level**					
No formal education	46(24.6)	40(27.6)	1	1	1
Primary	38(20.3)	45(31.0)	0.73(0.40,1.34)	1.07(0.49,2.34)	0.866
Secondary	64(34.2)	45(31.0)	1.24(0.69,2.19)	1.23(0.62,2.45)	0.556
Diploma and above	39(20.9)	15(10.4)	2.26(1.09,4.69)^a^	2.09(0.84,5.18)	0.112
**Family size**					
> 5	70(34.3)	65(42.2)	1	1	1
≤ 5	134(65.6)	89(57.8)	1.39(0.91,2.15)^a^	1.41(0.81,2.44)	0.226
**Geographical accessibility**					
Far(≥ 30 min)	46(22.5)	48(31.2)	1	1	1
Close (< 30 min)	158(77.5)	106(68.8)	1.56(0.97,2.49)^a^	1.42(0.78,2.58)	0.249
**Availability of Youth clubs**					
No	59(28.9)	107(69.5)		1	
Yes	145(71.1)	47(30.5)	5.59(3.54,8.84)^a^	5.06(2.92,8.77)^b^	< 0.001
**Ever had sexual partner/s**					
No	143(70.1)	124(80.5)	1	1	
Yes	61(29.9)	30(19.5)	1.76(1.07,2.90)^a^	1.72(0.90,3.30)	0.101
**Had a parental discussion on SRH issues**					
No	81(39.7)	112(72.7)	1		
Yes	123(60.3)	42(27.3)	4.05(2.58,6.36)^a^	3.25(2.01,5.67)^b^	< 0.001
**Participated in peer to peer education**					
No	61(29.9)	64(41.6)	1		
Yes	143(70.1)	90(58.4)	1.55(0.91,2.49)	1.12(0.83.1.87)	0.091
**Knowledge of SRH issues**					
Not knowledgeable	88(43.1)	84(54.5)	1	1	
Knowledgeable	116(56.9)	70(45.5)	1.58(1.04,2.4)^a^	1.46(0.84,2.53)	0.184

Key: 1: Reference category; *AOR* Adjusted odds ratio, *COR* Crude odds ratio

^a^statistically significant at *p*-value < 0.25, ^b^statistically significant at *p*-value < 0.05

The third model was designed particularly for rural adolescents. As a result, rural adolescents with adequate SRH knowledge were 2.9 times more likely than their counterparts to receive SRH services [AOR = 2.93; 95%CI: 1.94, 4.43]. Adolescents who lived in areas with functioning youth clubs were 4.2 times more likely to use SRH services than their counterparts[AOR = 4.23; 95%CI: 2.83,6.32]. Similarly, adolescents who had a parental discussion about SRH topics were 2 times more likely than those who did not [AOR = 2.09; 95%CI: 1.37, 3.20] to use SRH services. Geographical accessibility to nearby health facilities was also found as a significant predictor of SRH service uptake among rural adolescents. Those adolescents who had to travel less than 30 min to access a health facility were 2.1 times more likely to utilize SRH services than those who traveled 30 min and more[AOR = 2.1; 95%CI: 1.36,3.23] (Table 6).

**Table 6:** Determinants of SRH Service Utilization among rural adolescents in Guraghe Zone, Southern Ethiopia, 2020

Variable categories	SRH services utilization		COR(95%CI)	AOR(95%CI)	p-value
	Yes (%)	No (%)			
**Age( in years)**					
15–16	85(35.5)	214(43.1)	1		
17–19	136(61.5)	282(56.9)	1.21(0.88,1.68)^a^		
**Sex**					
Female	123(55.7)	305(61.5)	1	1	
Male	98(44.3)	191(38.5)	1.27(0.92,1.75)^a^	1.29(0.85,1.93)	0.228
**School enrolment status**					
No	13(5.9)	49(9.9)	1	1	
Yes	208(94.1)	447(90.1)	1.75(0.93,3.30)^a^	1.56(0.69,3.49)	0.283
**Educational status**					
No formal education	6(2.7)	24(4.8)	1	1	
Primary	88(39.8)	235(47.4)	1.49(0.59,3.79)	1.65(0.42,6.46)	0.468
Secondary	127(57.5)	237(47.8)	2.14(0.85,5.38)^a^	2.47(0.63,7.60)	0.193
**Current living arrangement**					
With husband/ wife	5(2.3)	29(5.8)	1		
With father only	7(3.1)	17(3.4)	2.39(0.65,6.71)^a^	2.12(0.71,4.01)	0.135
With mother only	17(7.7)	44(8.9)	2.24(0.74,5.74)^a^	2.01(0.65,4.12)	0.210
With bother parent	192(86.9)	406(81.9)	2.74(1.05,6.19)^a^	2.17(0.75,5.30)	0.123
**Mother’s education level**					
No Formal education	137(64.0)	261(54.5)	1	1	
Primary	36(16.8)	162(33.8)	0.72(0.48,1.64)	0.61(0.52,1.37)	0.532
Secondary	28(13.1)	43(8.9)	1.24(0.74,2.08)	1.27(0.67,2.40)	0.467
Diploma and above	13(6.1)	13(3.0)	1.91(0.86,4.22)^a^	1.74(0.66,3.59)	0.263
**Father’s educational level**					
No formal education	78(38.2)	174(38.5)	1	1	
Primary	72(35.3)	171(37.8)	0.94(0.64,1.38)	1.12(0.69,1.79)	0.641
Secondary	33(16.2)	87(19.3)	0.85(0.52,1.37)	0.82(0.46,1.44)	0.490
**Diploma and above**	21(10.3)	20(4.4)	2.34(1.20,4.57)^a^	1.77(0.78,4.02)	0.170
Family size					
> 5	125(56.6)	243(49.0)	1		
≤ 5	96(43.4)	253(51.0)	0.74(0.54,1.71)		
**Geographical accessibility**					
Far(≥ 30 min)	136(61.5)	398(80.2)	1	1	
Close (< 30 min)	85(38.5)	98(19.8)	2.54(1.79,3.60)^a^	2.10(1.36,3.23)^b^	0.001
**Availability of youth clubs**					
No	85(38.5)	364(73.4)	1	1	
Yes	136(61.5)	132(26.6)	4.41(3.15,6.18)^a^	4.23(2.83,6.32)^b^	< 0.001
**Had sexual partner/s**					
No	170(76.9)	411(82.9)	1	1	
Yes	51(23.1)	85(17.1)	1.45(0.98,2.14)^a^	1.45(0.88,2.37)	0.142
**Had a parental discussion on SRH issues**					
No	127(57.5)	382(77.0)	1		
Yes	94(42.5)	114(23.0)	2.48(1.77,3.48)^a^	2.09(1.37, 3.20)^b^	0.025
**Participated in peer to peer education**					
No	71(32.1)	219(44.2)	1		
Yes	150(67.9)	277(55.8)	1.67(1.19,2.33)^a^	1.50(0.98,2.29)	0.059
**Knowledge of SRH issues**					
Not knowledgeable	77(34.8)	318(64.1)	1	1	
Knowledgeable	144(65.2)	178(35.9)	3.34(2.39,4.66)^a^	2.93(1.94,4.43)	< 0.001

Key: 1: Reference category; *AOR* Adjusted odds ratio, *COR* Crude odds ratio

^a^statistically significant at *p*-value < 0.25, ^b^statistically significant at *p*-value < 0.05

## Discussion

One of the most important health indicators for young people's immediate and long-term SRH needs is the Adolescent Sexual and Reproductive Health (ASRH) program [7, 15]. This study aimed at comparing SHR service uptake and their determinants among adolescents in urban and rural settings. Overall, 39.5%( 95%CI: 36.5, 42.4) of adolescents used SRH services. This finding was comparable with studies conducted in Awabel district, Northwest Ethiopia (41.2%), and Jimma Zone, southwest Ethiopia(41.1%) [25, 31]. There was a significant difference in SRH service adoption between adolescents in rural areas (30.8%) and urban (56.9%) areas. The current study found that SRH service uptake among urban adolescents was higher than in a similar study conducted in Debre Birhan town, Northern Ethiopia(33.3%), and Adama town, Eastern Ethiopia (34.0%) [24, 32]. This disparity may be explained by the time difference between those studies in which adolescent health is being prioritized through enhancing peer-to-peer education at the school level and providing youth clubs in the community.

There was a significant difference between the two groups when it came to individual components of the SRH service. 43.6% of urban and 30.3% of rural adolescents received SRH information and education. As compared to studies conducted elsewhere in Ethiopia, these figures were lower [24, 26, 27]. The use of modern contraception was assessed by asking for at least one form of method-mix in the previous 12 months. Only 34.7% of adolescents (urban = 33.8% and rural = 30.3%) were received at least one type of contraceptive, which was lower than the Mini-EDHS 2019 survey (36.5%) [22] and studies conducted in the Awabel district(45.4%) [25], Gondar city (79.5%) [26], Gobba city (71.4%) [27], Mekele city (85.8%) [33] and Anchar district (39.3%) [34]. The current study in which significant segments of adolescents were unable to obtain the majority of SRH service. Inaccessibility of service delivery points may be a contributing factor in the current study area's low coverage of all SRH services; as a result, a concerted effort and collaboration among local government and non-government stakeholders are needed to make services more available to improve SRH service provision.

Residence, having a peer-to-peer education, having a parental discussion, the availability of a youth club, and being knowledgeable on SRH issues were identified as factors that influence the uptake of SRH services among the whole adolescents.

Urban adolescents had a 2.64 times greater chance of utilizing SRH services than rural adolescents. The possible justification is that urban adolescents may have access to a variety of SRH programs tailored to their age in close surroundings through youth clubs and socio-cultural and economic contexts, which might improve service uptake [18]. In Ethiopia, health care coverage is relatively high in urban than rural [35]. Furthermore, adolescents in urban areas are more likely to receive information about ASRH programs from health care providers and mass media such as radio and television, which may have contributed to ASRH service uptake. Based on the findings, we suggest that responsible bodies make a concerted effort to give careful credit to the ASRH needs of rural adolescents through behavioural change communication and the establishment of youth clubs.

According to the results, getting a parental discussion has a positive impact on ASRH service adoption among all adolescents, including urban and rural. Adolescents who had a parental discussion about ASRH matters were more likely to use ASRH services than those who did not. Numerous research in Africa, including Ethiopia, supplemented this [24–26, 33, 36]. This may be because, if adolescents were free to discuss ASRH topics with their parents, they would have developed more knowledge and insight about ASRH services, allowing them to practice them. To enhance adolescent–parent interaction on SRH issues, parents play a critical role. Adolescent sexual beliefs, attitudes, and behaviors were influenced by parent-adolescent sexual communication [37, 38]. According to a survey conducted in Ghana, adolescents who discuss SRH issues with their parents are more likely than other youths to delay initiation of sex and, once they do initiate sex, are more likely to use SRH services, such as contraceptive methods. Hence, rather than focusing solely on caring for the family, parents should be more nurtured by making themselves readily available to their youngsters.

Adolescents who engaged in peer education about ASRH issues had a higher likelihood of using the ASRH service. This is supported by similar studies done in Awabel district, Northern Ethiopia, Kenya, and Myanmar [25, 39, 40]. This might be because peer groups/friends are made up of people of similar ages or social groups, giving them the ability to share ideas and information about SRH problems with little to no restrictions. As a result, there would be more demand for and use of the ASRH service. Due to socio-cultural norms and taboos, economic inequality, or a lack of knowledge, adolescents in most societies find it difficult to obtain consistent and accurate information on SRH issues that affect them, such as sex, sexuality, drug use, reproductive health, HIV/AIDS, and STIs [41]. Information is almost always accessible, but it is presented in a way that is restrictive, judgmental, or unsuited to the values, views, and lifestyles of young people, and peer-to-peer education is critical in overcoming these stumbling blocks.

The current study found that adolescents who are knowledgeable about SRH issues are more likely to use ASRH services than their counterparts. Furthermore, knowledgeable rural adolescents had a higher likelihood of using SRH services. This is supported by a similar study done in northern Ethiopia [23]. This is reasonable because the more adolescents understand SRH, including its advantages, content, and service delivery points, the more likely they are to use the SRH services that are recommended. As a result, stakeholders must work together to improve adolescents' awareness of SRH resources through behavioral change communication strategies to increase ASRH service uptake.

The existence of a youth club in their immediate environment determines whether or not they use the ASRH service. Adolescents who claimed that there were youth clubs in their immediate environment were more likely to use SRH services than those who reported that there was no youth club. This is in line with research undertaken in African countries [28–30]. If youth clubs are accessible to adolescents, they may be able to increase SRH service adoption by increasing peer-to-peer dialogue, making young clients feel more relaxed and confident in seeking help [28, 42, 43]. As a result, concerned bodies' concerted efforts to expand such youth clubs in the zone, especially to rural (hard-to-reach) areas, have proven to be an effective strategy for enhancing SRH services uptake among adolescents.

The distance (perceived time spent traveling to health facilities) was found to be a significant predictor of rural adolescents' use of SRH services. Adolescents who had to travel less than 30 min to get to the health facility were 2.1 times more likely to use SRH services than those who had to travel more than 30 min. This is supported by studies conducted in Mozambique and Nigeria [44, 45]. This implies that outreach programs should be expanded to reach adolescents who reside far from health facilities.

This research has both strengths and limitations. This is the first study of its kind to compare the uptake of SRH services by adolescents in urban and rural settings and may have important policy implications for the further improvement of SRH services. Furthermore, adequate samples were taken to determine the contrast between the two groups. While every effort has been taken to reduce the study's flaws, readers should proceed with caution when interpreting the results. Respondents may be prone to social desirability biases, which may have contributed to underreporting of some SRH services, since that it involves some sensitive issues and was based on self-reports. Finally, there is a likelihood of recall bias because the adolescents in this study were inquiring about events that had already occurred.

## Conclusions

There was a significant difference in SRH service utilization between urban and rural adolescents. Residence, availability of youth clubs, taking part in peer education, having a parental discussion, and being knowledgeable on SRH issues were identified as significant determinants of SRH service uptake. Having parental discussion, geographical accessibility, and knowledge on SRH were significant predictors of SRH service uptake among rural adolescents. Since the majority of adolescents were enrolled in schools, schools should be an area of intervention to improve adolescents' knowledge of SRH services through mass media, community networks, and interpersonal/group communication. Furthermore, promoting parent-adolescent discussions, as well as peer-to-peer discussions at the family and school level, should be emphasized. Stakeholders in the education and health sectors need to strengthen their efforts to establish youth clubs in places where they do not yet exist, especially in rural schools.

## Supplementary Information

**Additional file 1. **

**Additional file 2. **

## Data Availability

The data used to support the findings of the current study can be obtained from the corresponding author on reasonable request via akliluhabte57@gmail.com.

## Abbreviations

AOR: Adjusted Odds Ratio
ASRH: Adolescent Sexual and Reproductive Health
EDHS: Ethiopian Demographic Health Survey
FMOH: Federal Ministry of Health
MLR: Multivariable Logistic Regression
ASRH: Adolescent Sexual And Reproductive Health
VCT: Voluntary HIV/AIDS Counseling, and Testing
WHO: World Health Organization
