# Supplementary material for: Disparities in sexual and reproductive health services utilization among urban and rural adolescents in southern Ethiopia, 2020: a comparative cross-sectional study
Source: BMC Public Health. 2022 Jan 31;22:203. doi: 10.1186/s12889-022-12634-x (PMC8802483; doi:10.1186/s12889-022-12634-x)
Supplement: Supplementary file 1 — Additional file 1. [file 12889_2022_12634_MOESM1_ESM.docx]

## Annex4:--Data collection tool to assess SRH service utilization and its associated factors among rural adolescents in Guraghe zone, Southern ethiopia, 2020

| Results of questionnaire: - | |
| --- | --- |
| 1. Completed [_____] | 1. Participant Refused [_____] |
| 1. Partially completed [_____] | **4.** |

| Respondents identification ______  Questionnaire Code______  **Instruction: Circle the appropriate answer** provided and where applicable writes the required responses in the spaces provided.  **SECTION 1: Background characteristics of respondents** | | | | | | | | | | |
| --- | --- | --- | --- | --- | --- | --- | --- | --- | --- | --- |
| S. no | Questions | | | Response | | | | | | Skip |
| **Part I: Identifications** | | | | | | | | | |  |
| 101 | Kebele Name | | |  | | | | | |  |
| 102 | Name of data collector | | |  | | | | | |  |
| 103 | Interview Starting time | | |  | | | | | |  |
| 104 | Finishing time | | |  | | | | | |  |
| **Part II: Socio-demographic and economic characteristics** | | | | | | | | | | |
| 201 | | How old are you? Age (in years) | 1. [___________]  2. I don’t know------------ | | | | | | |  |
| 202 | | What is your Marital status? | 1. Married | | | | | | 1. Divorced |  |
|  |  |  | 1. single | | | | | | 1. Widowed |  |
|  |  |  | 1. Separated due to work | | | | | |  |  |
| 203 | | What is your religion? | 1. Orthodox | | | | | | 1. Muslim |  |
|  |  |  | 1. Protestant | | | | | | 1. Catholic |  |
|  |  |  | 1. Other | | | | | |  |  |
| 204 | | To what ethnicity you belong to? | 1. Guraghe | | | | | | 1. Amhara |  |
|  |  |  | 1. Oromo | | | | | | 1. Siltei |  |
|  |  |  | 1. Hadiya | | | | | | 1. Others |  |
| 205 | | Where is your residence | 1. urban | | | | | | 2. Rural |  |
| 206 | | What is your educational level? | 1. No formal education | | | | | | 1. 1-8^th^ |  |
|  |  |  | 1. 9-12^th^ | | | | | | 1. College and above |  |
| 207 | | What is occupation of Your mother | 1. House wife | | | | | | 1. Merchant |  |
|  |  |  | 1. Farmer | | | | | | 1. Government employer |  |
|  |  |  | 1. Daily laborer | | | | | | 1. Others |  |
| 208 | | What is paternal educational status? | 1. No formal education | | | | | | 1. 1-8^th^ |  |
|  |  |  | 1. 9-12^th^ | | | | | | 1. College and above |  |
| 209 | | What is paternal present Occupation? | 1. Farmer | | | | | | 2.Government employee |  |
|  |  |  | 3. Merchant | | | | | | 4. Daily laborer |  |
|  |  |  | 5. other | | | | | | 6. No work |  |
| 210 | | How many members are there within the family? | [________________] | | | | | | |  |
| 211 | | How much is monthly income of the family in birr? | | | |  | | | |  |
| 212 | | How long does it take you to walk to reach nearby health facility from your home? | | | | Minuit/hours [_______]  I don’t know---------------- | | | |  |
| 213 | | Which health facilities you used while you are in need of SRH services | | | | 1. Health center 2. Hospital 3. Private clinics 4. Private pharmacies 5. Health posts | | | |  |
| 214 | | In your community, are there youth clubs? | | | | 1. Yes 2. No **if no skip to 301** | | | |  |
| 215 | | Do you participate in clubs that have been? | | | | 1. Yes 2. No | | | |  |
| **PARTIII: Assessment of Respondents’ attribute related to sexuality and reproductive health** | | | | | | | | | |  |
| 301 | Do you Ever had sexual partner/s | | | | 1. Yes 2. No, **if no skip to 307** | | | | |  |
| 302 | How many sexual partners you ever had | | | |  | | | | |  |
| 303 | Ever had of sexual intercourse | | | | 1. Yes 2. No, | | | | | **307** |
| 304 | Did you Use contraceptives during their first sexual intercourse | | | | 1. Yes 2. No | | | | |  |
| 305 | Do you Had sexual intercourse within the last 12 months | | | | 1. Yes 2. No | | | | |  |
| 306 | Frequency of sexual intercourse | | | | 1. Once 2. More than Once with the same partner 3. More than Once with a different partner | | | | |  |
| 307 | Do you Perceive that you are at risk towards HIV/AIDS infection | | | | 1. Yes 2. No | | | | |  |
| 308 | Ever had a parental discussion on SRH issues (n=1009) | | | | 1. Yes 2. No | | | | |  |
| 309 | Do you Counseled and Provided with modern contraception | | | | 1. Yes 2. No , | | | | | **310** |
| 310 | Contraceptive utilization by method mix | | | | 1. Oral contraceptives 2. Injectables 3. Condom 4. Implants 5. IUD 6. Others | | | | |  |
| 311 | Have you ever drink alcohols? | | | | 1. Yes 2. No , | | | | | **315** |
| 312 | Have you drink alcohol within the last 12 months | | | | 1. Yes 2. No | | | | |  |
| 313 | Have you drink alcohol within the last 6 months | | | | 1. Yes 2. No | | | | |  |
| 314 | How frequently do you drink alcohol? | | | | 1. Almost every day 2. At least once a week 3. At least once a month 4. At least once a year 5. Ceased currently(if he/she ceased for the last 3 months) | | | | |  |
| 315 | Have you ever chewed the Khat? | | | | 1. Yes 2. No , | | | | | **319** |
| 316 | Have you chew ‘Khat’ within the last 12 months | | | | 1. Yes 2. No | | | | |  |
| 317 | Have you chew ‘khat’ within the last 6 months | | | | 1. Yes 2. No | | | | |  |
| 318 | How frequently do you chew ‘khat’? | | | | 1. Almost every day 2. At least once a week 3. At least once a month 4. At least once a year 5. Ceased currently(if he/she ceased for the last 3 months) | | | | |  |
| 319 | Have you ever smoke cigarette? | | | | 1. Yes 2. No , | | | | | 401 |
| 320 | Have you smoke within the last 12 months | | | | 1. Yes 2. No | | | | |  |
| 321 | Have you smoke within the last 6 months | | | | 1. Yes 2. No | | | | |  |
| 322 | How frequently do you smoke? | | | | 1. Almost every day 2. At least once a week 3. At least once a month 4. At least once a year 5. Ceased currently(if ceased for the last 3 months) | | | | |  |
| **Part-Iv: Assessment Of Knowledge Of Adolescents Towards SRH Issues** | | | | | | | | | |  |
| 401 | Do you Ever heard about SRH? | | | | | | 1. Yes 2. No | | | **403** |
| 402 | What are your Source of information? | | | | | | 1. From school 2. Radio 3. Television 4. Social media 5. Family members | | |  |
| 403 | Can you mention at least one SRH service that should be delivered to an adolescent? | | | | | | 1. Yes 2. No | | |  |
| 404 | Do you Know delivery points for SRH services? | | | | | | 1. Yes 2. No | | |  |
| 405 | Do you Know SRH service provider? | | | | | | 1. Yes 2. No | | |  |
| 406 | Do you Know the reasons for unintended pregnancy | | | | | | 1. Yes 2. No | | |  |
| 407 | Know at least one way of avoiding pregnancy | | | | | | 1. Yes 2. No | | | 409 |
| 408 | What are those mechanisms | | | | | | 1. Abstinence 2. Condom use 3. Pills 4. Injectables 5. Others | | |  |
| 409 | Do you Know at least one type of STI | | | | | | 1. Yes 2. No | | |  |
| 410 | Know the mode of transmission of STI | | | | | | 1. Yes 2. No | | |  |
| 411 | Can mention at least one mechanism of STI prevention? | | | | | | 1. Yes 2. No | | |  |
| 412 | Do you Know the place where STI case management are availed? | | | | | | 1. Yes 2. No | | |  |
| 413 | Do you Know about the benefits of contraceptive methods? | | | | | | 1. Yes 2. No | | |  |
| 414 | Do you Know at least one type of contraceptive? | | | | | | 1. Yes 2. No | | | 501 |
| 415 | Can you mention at least one of the method-mix? | | | | | | 1. Condom 2. Oral contraceptives 3. Injectables 4. Implants 5. IUD 6. Others | | |  |
| **PART-VI :- ASSESSMENT OF SRH SERVICE UTILIZATION** | | | | | | | | | |  |
| Sr.no | **Contents of service packages in PCC** | | | | | | | **Responses** | |  |
| 501 | Do you get voluntary counseling and testing(VCT) service for HIV | | | | | | | 1. Yes 2. No | |  |
| 502 | Do you Provided with of comprehensive SRH education | | | | | | | 1. Yes 2. No | |  |
| 503 | Do you get STI Prevention and care | | | | | | | 1. Yes 2. No | |  |
| 504 | Do you get counselling and modern contraceptive provision? | | | | | | | 1. Yes 2. No | |  |
| 505 | Provided with counseling and provision of safe abortion care | | | | | | | 1. Yes 2. No | |  |
| 506 | In the last 12 months, have you engaged in peer-to - peer education in your school or village? | | | | | | | 1. Yes 2. No | |  |
| 507 | Do you get advice on violence against women and girls (VAWG) prevention? | | | | | | | 1. Yes 2. No | |  |
| 508 | Do you get information on harmful traditional practices (HTPs)? | | | | | | | 1. Yes 2. No | |  |

**THANK YOU VERY MUCH!!!**
